# Calcification in free-living coralline algae is strongly influenced by morphology: Implications for susceptibility to ocean acidification

**DOI:** 10.1038/s41598-021-90632-6

**Published:** 2021-05-27

**Authors:** Nadine Schubert, Laurie C. Hofmann, Antonella C. Almeida Saá, Anderson Camargo Moreira, Rafael Güntzel Arenhart, Celso Peres Fernandes, Dirk de Beer, Paulo A. Horta, João Silva

**Affiliations:** 1 CCMAR - Center of Marine Sciences, University of Algarve, Campus Gambelas, 8005-139 Faro, Portugal; 2 Phycology Laboratory, Botany Department, Center for Biological Sciences, Federal University of Santa Catarina, Campus Trindade, Florianopolis, 88010-970 Brazil; 3 Microsensor Group, Max Planck Institute for Marine Microbiology, Celsiusstrasse 1, 28359 Bremen, Germany; 4 Graduate Program in Oceanography (PPGOCEANO), Center for Physical and Mathematical Sciences, Federal University of Santa Catarina, Campus Trindade, Florianopolis, 88010-970 Brazil; 5 Porous Media and Thermophysical Properties Laboratory (LMPT), Mechanical Engineering Department, Federal University of Santa Catarina, Campus Trindade, Florianopolis, 88010-970 Brazil; 6 Present Address: Marine Aquaculture Group, Alfred Wegener Institute Helmholtz Center for Polar and Marine Research, Am Handelshafen 12, 27570 Bremerhaven, Germany; 7 Present Address: Institute of Oceanological Research (IIO), Autonomous University of Baja California, Km 106. Carretera Tijuana-Ensenada, 22860 Baja California, Ensenada, Mexico

**Keywords:** Plant sciences, Ecology, Environmental sciences, Ocean sciences

## Abstract

Rhodolith beds built by free-living coralline algae are important ecosystems for marine biodiversity and carbonate production. Yet, our mechanistic understanding regarding rhodolith physiology and its drivers is still limited. Using three rhodolith species with different branching morphologies, we investigated the role of morphology in species’ physiology and the implications for their susceptibility to ocean acidification (OA). For this, we determined the effects of thallus topography on diffusive boundary layer (DBL) thickness, the associated microscale oxygen and pH dynamics and their relationship with species’ metabolic and light and dark calcification rates, as well as species’ responses to short-term OA exposure. Our results show that rhodolith branching creates low-flow microenvironments that exhibit increasing DBL thickness with increasing branch length. This, together with species’ metabolic rates, determined the light-dependent pH dynamics at the algal surface, which in turn dictated species’ calcification rates. While these differences did not translate in species-specific responses to short-term OA exposure, the differences in the magnitude of diurnal pH fluctuations (~ 0.1–1.2 pH units) between species suggest potential differences in phenotypic plasticity to OA that may result in different susceptibilities to long-term OA exposure, supporting the general view that species’ ecomechanical characteristics must be considered for predicting OA responses.

## Introduction

Marine coastal ecosystems formed by free-living coralline algae that cover 30–100% of the seafloor, so-called rhodolith or maërl beds, are distributed worldwide and have long been known to be important biodiversity hot-spots and major carbonate-producing ecosystems^1,2^. Because of their importance for biodiversity, in several regions of the world they are considered critical habitats for marine conservation and protected by a range of directives, regulations and conventions^2^. Also, more recently it has been pointed out that these habitats may figure more prominently in the global CaCO_3_ production than currently recognized^3–5^. This seems reasonable, when considering that in the Southwestern Atlantic alone rhodolith beds extend almost continuously for over 4.000 km along the Brazilian coast (2°N to 27°S)^6^, covering an estimated area of 230,000 km^2^^7^.

Like many other marine ecosystems, these habitats are currently impacted by environmental pressures associated with climate change, either due to direct effects on rhodoliths or indirect effects due to altered species interactions^8–15^. As calcifying organisms, rhodoliths are especially prone to be negatively impacted by ocean acidification (OA) and the little evidence so far available suggests a large variability in their responses^16^. In combination with our limited mechanistic understanding of their calcification mechanism, its regulation and potential species-specific differences, this makes it difficult to anticipate potential future impacts on these organisms and hence, the ecosystems they build—a point widely stressed as an important tool to improve predictive power of OA studies^17–20^.

Current evidence shows that coralline algae exhibit species-specific differences in their ability to physiologically control the calcification process, which in turn seems to dictate their resistance to OA^21–26^. Previous studies demonstrated that this is related to differences in the species’ capacity to elevate the pH of the calcifying fluid^21–27^ and within the diffusive boundary layer (DBL) at the algal surface^24,28^. The build-up of a pH gradient within the DBL, which exhibits a linear relationship with the pH of the calcifying fluid^24^, and its magnitude are determined by species’ metabolic activity, DBL thickness and seawater pH^24,29–32^. Generally, increased DBL thickness favors larger pH gradients, with the former being inversely related to water flow velocity^24,29,30^. Water flow velocity can vary largely in the natural environment of the algae, but at a microscale, it also varies depending on the morphology of the algae (i.e. thallus topography) that can create low-flow microenvironments^24,33^. While the effects of water flow velocity on coralline algal calcification and species’ OA responses have been demonstrated in previous studies^24,29,30^, so far the role of morphology for species’ calcification rates and the implications for the species’ OA responses are not well understood.

Rhodoliths are a morphologically highly variable group^34,35^ and to date, only the study by Hurd et al.^29^ on the temperate rhodolith *Sporolithon durum* has provided evidence of the effects of rhodolith surface topography on DBL thickness and microscale pH dynamics at the thallus surface. It showed that the “bumpy” thallus topography creates microenvironments with thicker DBLs in-between “bumps”, where thallus surface pH is greatly increased, when compared to the tip of the “bumps”. This suggests an important role for morphology in these calcifiers, as the build-up of a pH gradient has a strong effect on the calcification process^24,30^.

Here we hypothesize that besides inherent physiological differences among rhodolith species, morphology plays an important role in determining species’ capacity to modulate thallus surface pH and hence, calcification rates, which in turn may lead to potential differences in their responses to OA. To test this hypothesis, the goals of this study were to determine (1) whether there is a relationship between rhodolith branching morphology, DBL thickness and associated concentration gradients at the thallus surface, (2) whether the resulting algal surface pH dynamics have an influence in species’ light- and dark calcification rates, and (3) whether morphology-associated diurnal pH fluctuations affect the species’ response to short-term (shock-) exposure to lower seawater pH conditions.

## Results

**Figure 1:**
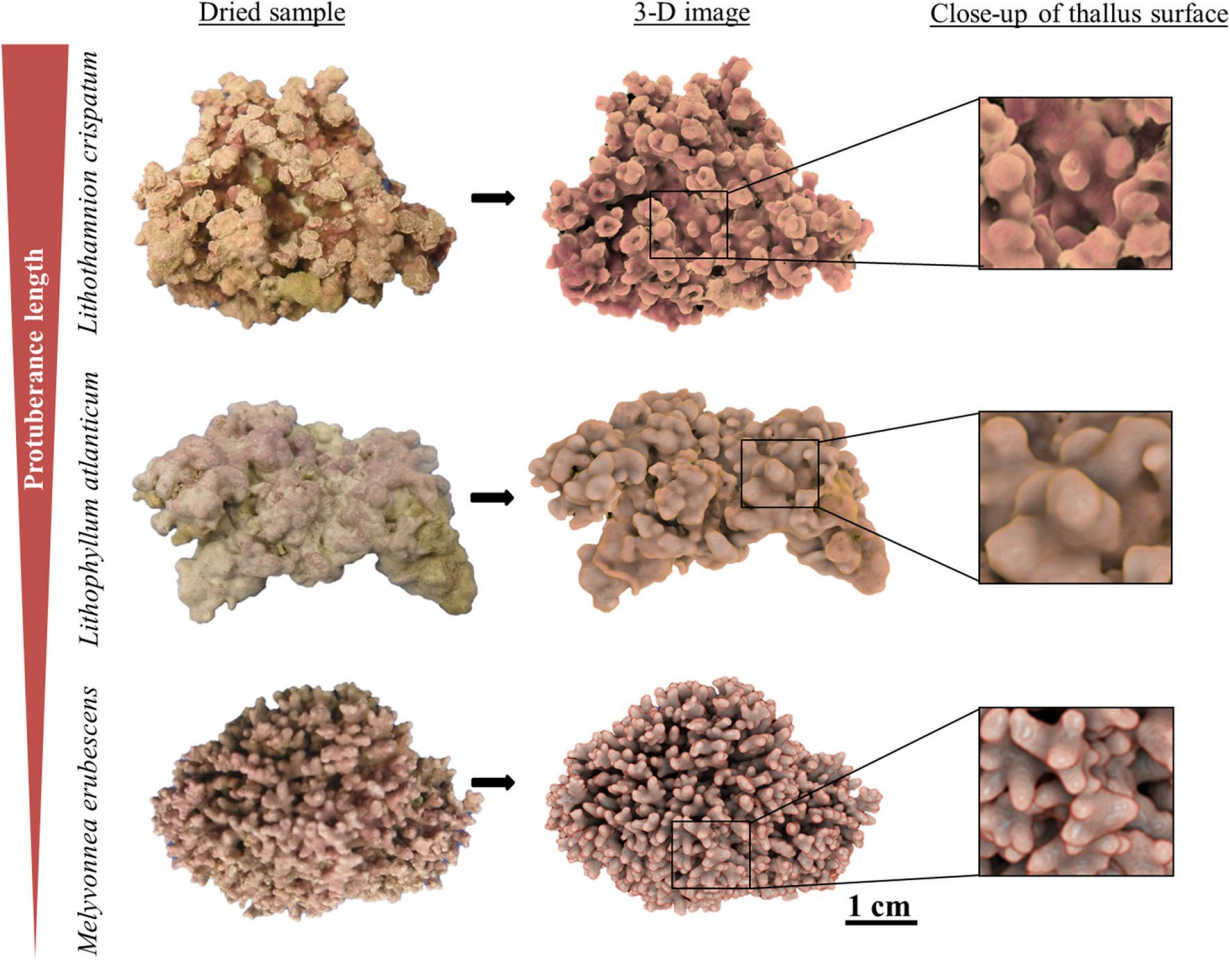
Morphology of the studied rhodolith species: *Lithothamnion crispatum* shows a lumpy to fruticose morphology and long cylindrical protuberances (~ 15 mm length, 2 mm diameter) ^36^ flaring distally into curly margined, with funnel-like or cup-shaped tips, *Lithophyllum atlanticum* has a smooth or warty to lumpy surface with shorter protuberances (5–10 mm length, 3.5 mm diameter) ^37^, and *Melyvonnea erubescens* with its fruticose to warty morphology exhibits branched and even shorter protuberances (1.5–4 mm length, 1–2 mm diameter) ^38^. Left row shows images of dried rhodoliths, middle row their respective 3-D images, obtained by X-ray Micro-CT scans, and right row a close-up on the thallus surface. The relative difference in protuberance lengths between species is indicated on the left.

**Figure 2:**
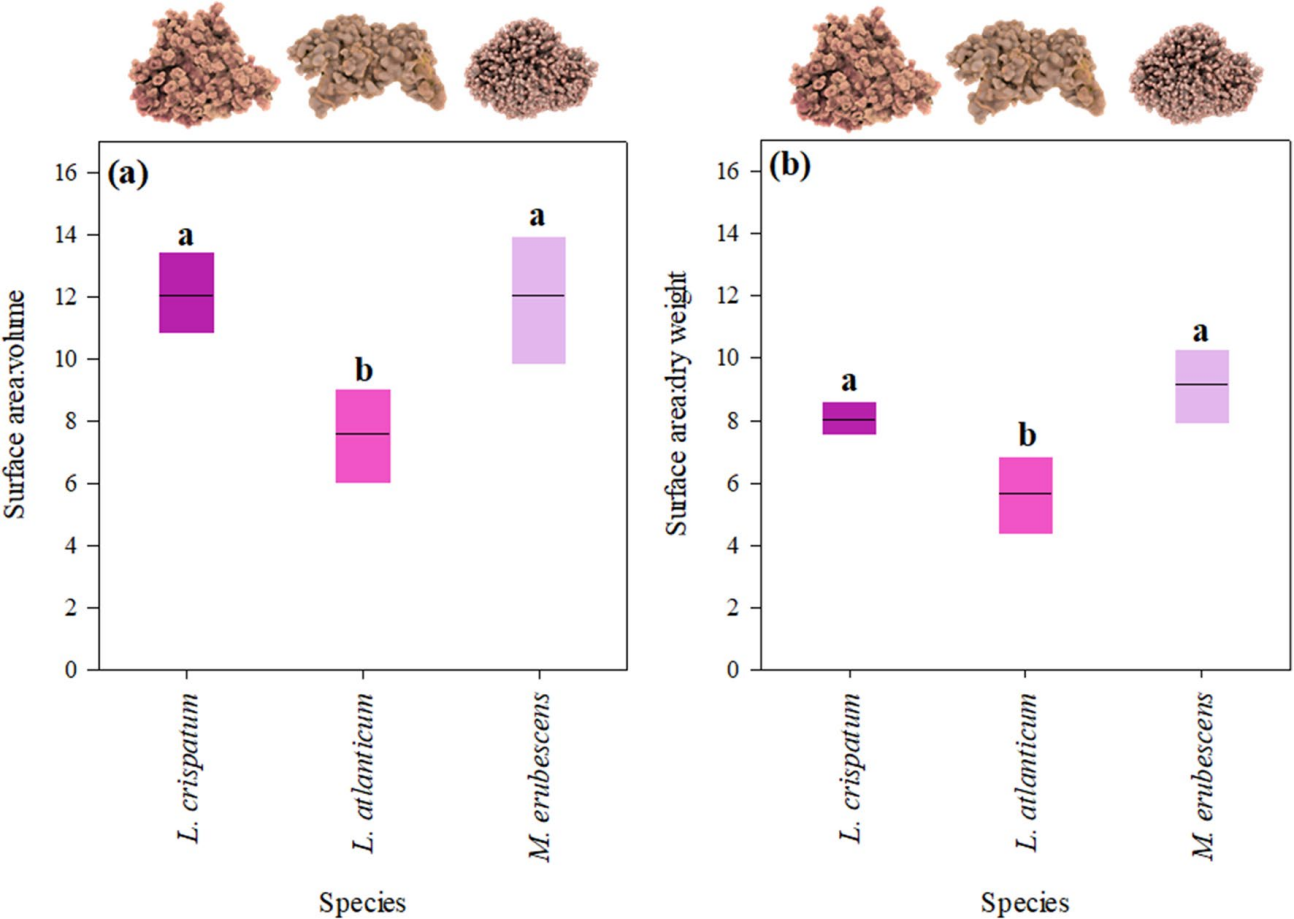
Comparison of the morphological complexity of *Lithothamnion crispatum*, *Lithophyllum atlanticum* and *Melyvonnea erubescens*, indicated by **a** surface area to volume ratios and **b** surface area per dry weight ratios. Data are given as mean ± SE (n = 5 per species) and significant differences between species (ANOVA, Newman-Keuls, *p* < 0.05) are indicated by different letters.

The morphological differences of the studied rhodolith species are mainly related to the number and size of protuberances (or branches) that result in highly distinctive thallus surface topographies (Fig. 1). Quantitatively, this was reflected in species-specific differences in surface area per volume (SA/V; ANOVA, *p* = 0.0004) and dry weight ratios (SA/DW; ANOVA, p = 0.0031). Two of the species, *Lithothamnion* *crispatum* and *Melyvonnea* *erubescens*, exhibited similar SA/V ratios and SA/DW, while those ratios were significantly lower in *Lithophyllum atlanticum* (Fig. 2).

**Figure 3:**
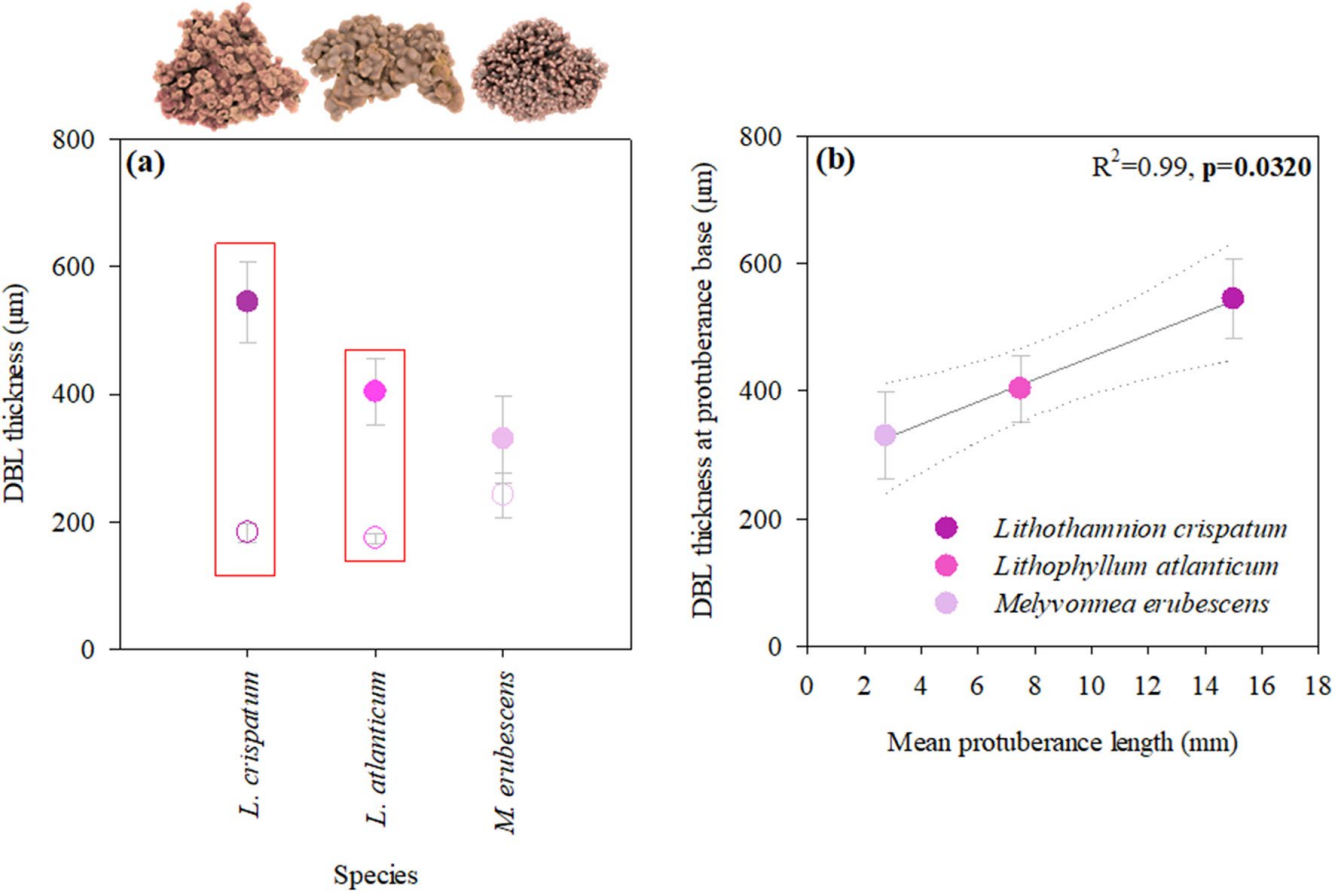
Influence of rhodolith morphology on diffusive boundary layer (DBL) thickness. (**a**) Comparison between DBL thickness at protuberance tips (empty circles) vs. bases (filled symbols) and (**b**) the linear relationship between rhodolith protuberance length (mean reported values) and DBL thickness at the protuberance base (dotted lines represent 95% confidence bands). Data represent mean ± SE (n = 3 per species and location) and red boxes in (**a**) indicate significant differences between tip and base in the respective species (ANOVA, Newman Keuls, *p* < 0.05).

The effect of species’ morphological differences on microscale biochemical dynamics at the algal thallus surface was assessed through microelectrode oxygen and pH measurements, which indicated the presence of discrete microenvironments in-between protuberances that exhibited differences in DBL thickness and the concentration gradients within. Comparing the DBL thickness at protuberance tips and bases, no significant differences were found between species, but between protuberance tips and bases (Table 1). The magnitude of these differences varied between species, with significantly thicker DBLs at the bases compared to the tips in *L. crispatum* (Fig. 3a; ANOVA, *p* = 0.0002). Significantly thicker DBLs at the protuberance bases were also found in *L. atlanticum* (ANOVA, *p* = 0.0068), though the differences were smaller than in the former species (Fig. 3a). *Melyvonnea erubescens* exhibited a similar pattern, though the differences between protuberance tips and bases were much smaller than in the other species and statistically insignificant (Fig. 3a ANOVA, *p* = 0.2097). These species-specific differences were directly related to the species’ protuberance length, as shown by the significant increase in DBL thickness at the protuberance bases with their respective lengths (Fig. 3b).

**Table 1:** Summary of the results of two-way ANOVA analyses, testing for the effects of species and their respective morphology (location- protuberance tip vs. base) on DBL thickness and the associated biochemical gradients. Significant differences (*p* < 0.05) are indicated in bold.

Parameter	Morphology (protuberance tip vs. base)					
	Species		Location		Species x Location	
	F	*p*-value	F	*p*-value	F	*p*-value
*DBL thickness*	1.38	0.2678	36.00	< 0.0001	3.99	0.0299
*Oxygen flux*						
Light	16.28	0.0004	11.31	0.0056	4.22	0.0410
Dark	7.24	0.0087	0.74	0.4055	1.36	0.2927
*ΔpH*						
Light	8.96	0.0036	59.67	< 0.0001	0.40	0.6770
Dark	18.40	0.0016	31.88	0.0008	8.78	0.0124

**Figure 4:**
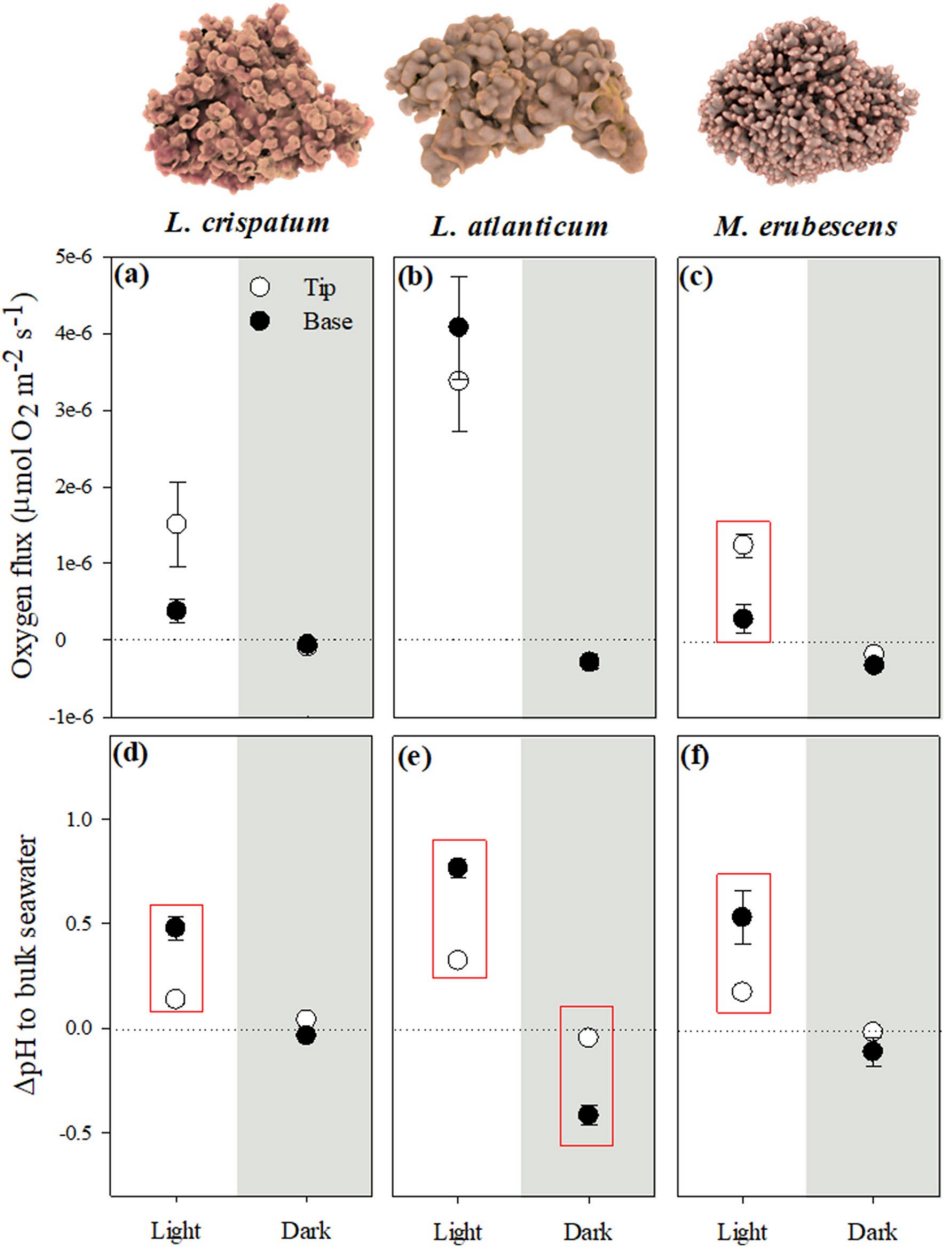
Effects of differences in rhodolith thallus surface topography (protuberance tip vs. base) on microscale oxygen and pH dynamics under light and dark conditions. (**a**–**c**) Species’ gross photosynthetic and respiratory rates and (**d**–**f**) the pH difference between thallus surface and bulk seawater. Data are shown as mean ± SE (n = 3) and red boxes indicate significant differences between tip and base for the respective species and light condition (ANOVA, Newman Keuls, *p* < 0.05).

The differences in DBL thickness between species were accompanied by differences in the associated microscale oxygen fluxes and pH dynamics (see Supplementary Fig. S1 online). Oxygen fluxes were significantly different between rhodolith species under light and dark conditions (Table 1), with *L. atlanticum* exhibiting the highest values in light, and together with *M. erubescens* the lowest values in darkness (Fig. 4a–c). Differences between species were also found when comparing the oxygen fluxes of protuberance tips vs. bases in light (Table 1). While *L. atlanticum* did not exhibit significant differences in light oxygen fluxes (Fig. 4b; ANOVA, *p* = 0.661), in both, *L. crispatum* and *M. erubescens*, the oxygen fluxes at the tips were higher than at the bases, though these differences were significant only in the latter species (Fig. 4a, c; ANOVA, *L. crispatum*, *p* = 0.0554, *M. erubescens*, *p* = 0.0099). In contrast, in darkness no differences in oxygen fluxes between the species’ protuberance tips and bases were found (Fig. 4a–c; Table 1).

Significant differences were also found between species and between protuberance tips and bases in both, light and dark conditions, regarding the pH dynamics at the algal thallus surface (Table 1; see Supplementary Fig. S1 online). In all species, significantly higher pH values were recorded under light conditions at the protuberance bases (~ 0.5–0.8 units higher than bulk seawater), compared to the tips (~ 0.2–0.3 units higher than bulk seawater) (Fig. 4d–f). In darkness, only *L. atlanticum* exhibited significantly lower pH values at the protuberance base (0.4 units lower than bulk seawater; ANOVA, p = 0.0005), while the pH at its protuberance tips, as well as at the tips and bases of the other two species, were not different from bulk seawater (Fig. 4d–f). Altogether, this indicated that *L. atlanticum* experienced high diurnal pH fluctuations at the thallus surface, ranging from 0.37 to 1.18 pH units at protuberance tips and bases, respectively, while the magnitude of fluctuations was much lower in the other two species. The pH_DBL_ in *Melyvonnea erubescens* exhibited a diurnal pH variability of 0.19–0.64 pH units, while it varied between 0.1 and 0.51 pH units in *L. crispatum* at protuberance tips and bases, respectively.

**Figure 5:**
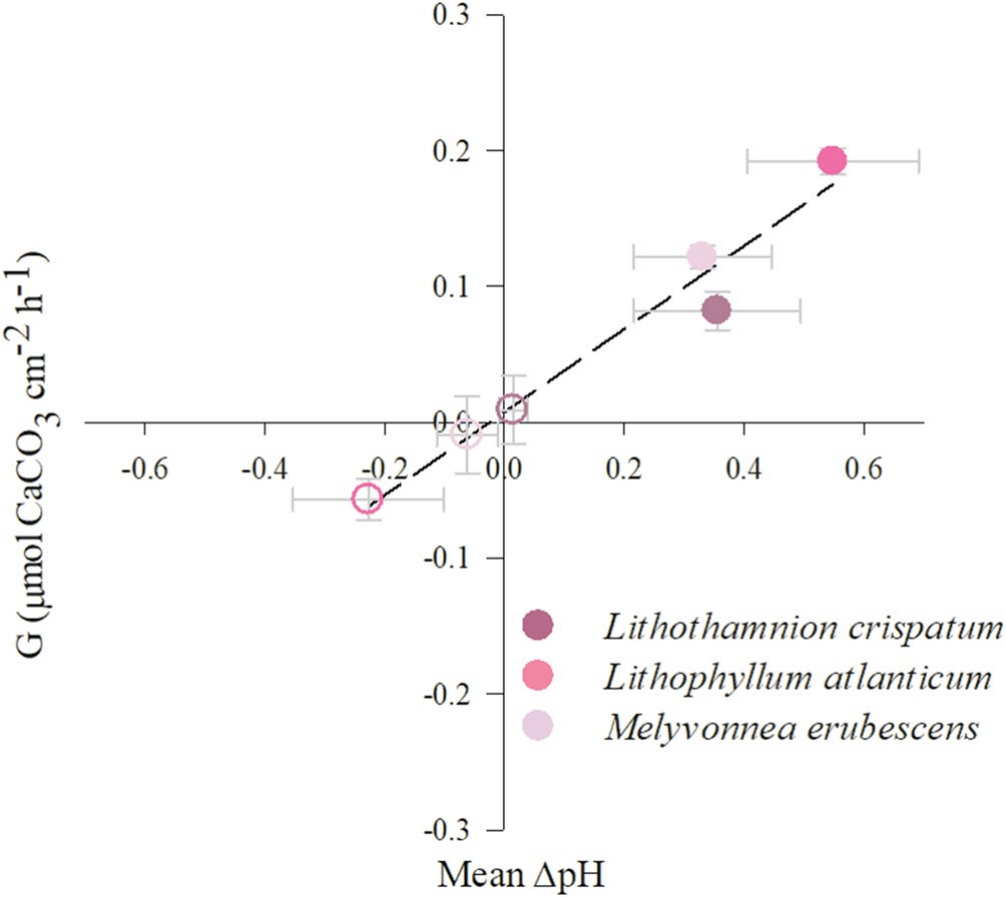
Linear relationship between light and dark ΔpH of the algal thallus surface compared to bulk seawater (mean between protuberance tip and base) and rhodolith light (filled circles) and dark (empty circles) calcification rates (R^2^ = 0.96, *p* = 0.0006).

The species-specific microscale oxygen and pH dynamics within the DBL at the thallus surface were consistent with the species’ metabolic and calcification rates, measured in the organisms as a whole. Between the three studied species, *L. atlanticum* expressed the highest metabolic rates (photosynthesis and dark respiration) (Table 2), agreeing with the recorded oxygen fluxes at the thallus surface (Fig. 4b). Furthermore, the species’ differences in calcification were consistent with the microscale pH dynamics, as evidenced by the significant direct relationship between the pH difference (mean of protuberance tips and bases) at the algal thallus surface (ΔpH_DBL_), compared to the bulk seawater, and the species’ light and dark calcification rates (Fig. 5). Under light conditions, the mean pH_DBL_ value of *L. atlanticum* was 0.55 units higher than the pH of the overlying bulk seawater and 0.33–0.36 units higher in the other two species, consistent with their differences in light calcification rates (Fig. 5; Table 2). Further, the mean pH_DBL_ in the dark was 0.23 units lower than the bulk seawater in *L. atlanticum*, agreeing with the species’ negative dark calcification rate, indicative for CaCO_3_ dissolution (Table 2). In contrast, in *L. crispatum* and *M. erubescens* the mean pH_DBL_ in darkness were 0.0009 pH units higher and 0.06 lower, respectively, associated with lower, but positive calcification in the former species, while *M. erubescens* also exhibited negative dark calcification rates (Table 2). Thus, between the three species, *L. atlanticum*, which exhibited the highest metabolic rates and consequently the highest and lowest pH at the algal surface under light and dark conditions, respectively (Fig. 4b, e), also expressed the highest light and lowest dark calcification rate (Fig. 5, Table 2).

**Table 2:** Metabolic and calcification rates of different rhodolith species under saturating light conditions, normalized by surface area (P_max_- maximum gross photosynthesis and DR- dark respiration, in µmol O_2_ cm^-2^ h^-1^; G_Light_ and G_Dark_- light and dark calcification, in µmol CaCO_3_ cm^-2^ h^-1^; G_Net_ – daily net calcification, in µmol CaCO_3_ cm^-2^ day^-1^, considering 14 h light:10 h dark). Data shown are mean ± SE (n = 5 per species). Statistical results of one-way ANOVAs, testing for differences between species, are given and indicated by superscript letters (Newman-Keuls post-hoc).

Parameter	*L. crispatum*	*L. atlanticum*	*M. erubescens*	F	*p*-value
P_max_	0.50 ± 0.03^a^	0.61 ± 0.02^b^	0.52 ± 0.03^a^	5.11	0.0269
DR	0.10 ± 0.02^a^	0.18 ± 0.01^b^	0.14 ± 0.01^ab^	7.68	0.0082
G_Light_	0.12 ± 0.01^a^	0.19 ± 0.01^b^	0.08 ± 0.01^c^	28.9	< 0.0001
G_Dark_	0.04 ± 0.01^a^	− 0.06 ± 0.01^b^	− 0.01 ± 0.03^ab^	10.23	0.0048
G_Net_	2.08 ± 0.20^a^	2.10 ± 0.32^a^	0.99 ± 0.15^b^	7.13	0.0205

**Figure 6:**
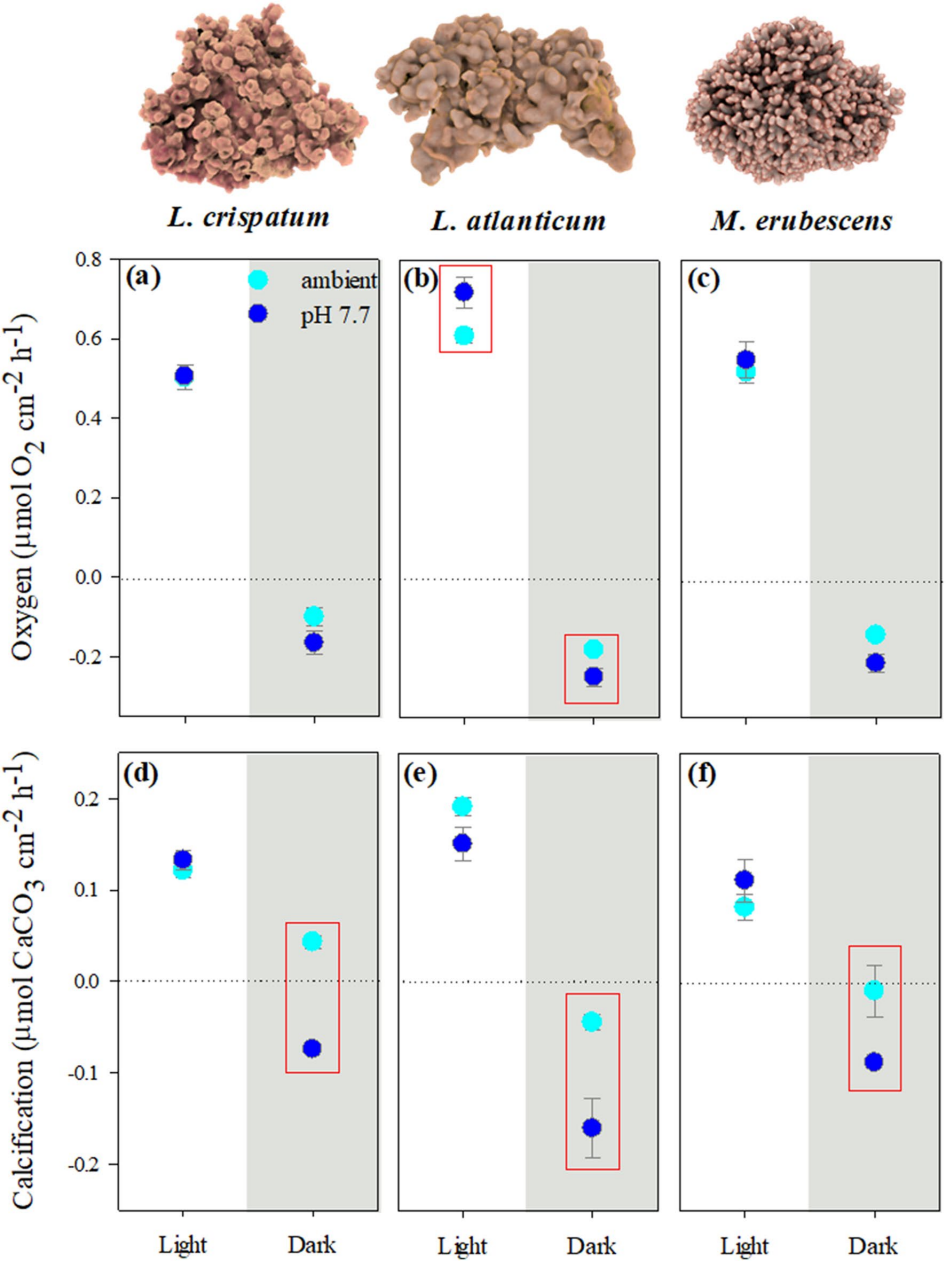
Rhodolith metabolic and calcification responses to ambient and low seawater pH. (**a**–**c**) Maximum gross photosynthesis and dark respiration and (**d**–**f**) light and dark calcification rates. Data are shown as mean ± SE (n = 5) and red boxes indicate significant differences between the two pH conditions (ANOVA, Newman Keuls, *p* < 0.05).

To study the potential influence of morphology and the aforementioned associated differences in biochemical gradients at the thallus surface on the OA responses of the species, the rhodoliths were exposed to a sudden decline in seawater pH (pH 7.7). General significant effects of this OA treatment were detected in dark respiration and dark calcification, but not in photosynthesis and light calcification (Table 3). At the species level, only *L. atlanticum* responded with a significant increase in gross photosynthesis (ANOVA, *p* = 0.031) and dark respiration (ANOVA, *p* = 0.0017), while all species showed a strong effect of lower pH on dark calcification, resulting in negative values that indicated net dissolution (Fig. 6, Table 3). In the case of *L. crispatum* and *M. erubescens*, their dark calcification rates under low-pH declined to the level of the negative dark calcification rate expressed by *L. atlanticum* under ambient seawater pH (ANOVA, *p *> 0.05), while in the latter the negative dark calcification rates under low-pH were significantly higher than in the other two species (Fig. 6d–f; ANOVA, *p* < 0.05).

**Table 3:** Summary of the results of two-way ANOVA analyses, testing for differences in the species’ response to short-term OA-exposure (ambient vs. low seawater pH) (P_max_- maximum gross photosynthesis, DR- dark respiration, G_Light_ and G_Dark_- light and dark calcification rates). Significant differences (*p* < 0.05) are indicated in bold.

Parameter	OA-experiment					
	Species		pH		Species x pH	
	F	*p*-value	F	*p*-value	F	*p*-value
P_max_	12.24	0.0003	2.65	0.1188	1.50	0.2455
DR	6.02	0.0086	16.35	0.0006	1.60	0.2262
G_Light_	11.75	0.0005	0.05	0.8261	2.90	0.0796
G_Dark_	20.31	< 0.0001	67.21	< 0.0001	0.90	0.4231

## Discussion

**Figure 7:**
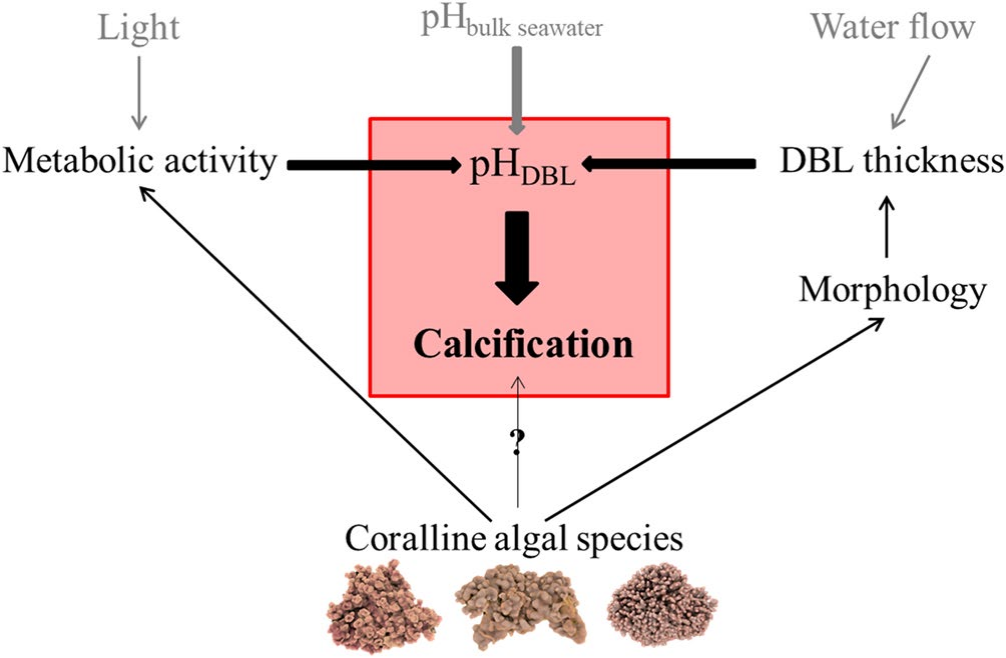
Schematic overview of the relationship between the pH within the DBL (pH_DBL_) and coralline species’ calcification rates, as well as the biotic (black) and abiotic factors (grey) affecting directly (thick arrows) and indirectly (thin arrows) the pH_DBL_ and hence, calcification. The influence of species-specific differences in the calcification mechanism cannot be excluded, but further information is required to infer about the effect on species’ calcification rates.

This study expands our understanding regarding the factors that regulate rhodolith calcification, showing that morphology and rhodolith species’ metabolism are critical factors determining diurnal fluctuations in thallus surface pH and consequently, species’ light and dark calcification rates (Fig. 7). While the species–specific magnitudes of daily pH fluctuations did not result in differences in species’ responses to short-term OA exposure, they suggest potential differences in phenotypic plasticity and hence, susceptibilities to long-term OA exposure.

The morphological differences of the studied rhodolith species are mainly related to the size of the protuberances (or branches), resulting in distinguished algal thallus surface topographies (see Fig. 1). Those were found to exhibit a strong effect on the DBL, showing an increased DBL thickness in-between protuberances, which is consistent with previous studies in the temperate rhodolith *Sporolithon durum*^29^ and in undulated *Macrocystis pyrifera* blades (undulation apex vs. within undulation)^33^. As shown in the latter study, topographic depressions, like those created by protuberances in rhodoliths, create low-flow microhabitats that lead to an increase of DBL thickness. This explains the species-specific increase in DBL thickness in-between protuberances as a direct result of the species’ protuberance lengths (Fig. 3). This effect of thallus topography on DBL thickness and in turn the concentration gradients within is a feature well documented in macroalgae^29,33^. However, our data also suggest that DBL thickness was not the sole factor defining the strength of the associated biochemical gradients. *Lithophyllum atlanticum*, which ranked second in protuberance length and DBL thickness, expressed the largest oxygen gradient and also the strongest pH gradient within the DBL (pH_DBL_). This was consistent with the higher metabolic rates of this species, compared to the others (Table 2), as the oxygen gradient within the DBL is a result of algal photosynthesis, respiration and DBL thickness. Further, it is linked directly to the pH dynamics, due to the uptake and release of dissolved inorganic carbon in light and dark ^32,39,40^. Altogether, this supports the general notion that both oxygen and pH gradients not only depend on DBL thickness, but also on the species metabolic activity^30,40^. Successively, this seemed to create a feedback loop, having a direct influence on the rhodolith calcification process, as shown by the significant direct relationship between the mean pH at the rhodolith thallus surface and the calcification rates under both light and dark conditions (Fig. 5). Similarly, a relationship between the pH dynamics at the algal thallus surface and species’ calcification rate had also been reported recently in *Sporolithon durum*^24^. The assertion appears particularly reasonable when considering that calcification in coralline algae takes place close to the thallus surface^41^ and that the pH at the calcification site shows a direct linear relationship with the pH_DBL_^24^. Interestingly and contrary to what would be expected, there was the slightly higher mean ΔpH_DBL_ in darkness in *L. crispatum* (0.0009), which resulted from higher ΔpH_DBL_ at the protuberance tips (0.04), compared to the bulk seawater, while at the bases the ΔpH_DBL_ was lower (− 0.036). We do not have an explanation for the higher ΔpH_DBL_ at the tips in darkness, though a recent study on Artic rhodoliths reported similar findings^28^. The authors suggested that it might be due to a reduction in respiratory CO_2_ release because of light-independent carbon fixation.

The rhodolith species exhibited significant differences in their metabolic rates (Table 2), which might be an inherent feature, but it could also be argued that these differences relate, at least partially, to light attenuation caused by the protuberances, increasing with protuberance length and resulting in less light reaching the respective bases, as suggested by Burdett et al.^42^. These authors found higher effective quantum yields and lower non-photochemical quenching at the protuberance bases, compared to the tips, in the rhodolith *Lithothamnion glaciale*, indicating lower light levels at the base. Yet, in our study, no differences in the light intensities measured with a light microsensor at the protuberance tips and their respective bases of the species were found (see Supplementary Fig. S2 online). A reason for this inconsistency could be the high variability between our measurements and insufficient replication, as *M. erubescens* showed a high variability and in some measurements the light intensity at the protuberance base was ~ 20% lower compared to the tip, consistent with the found significant differences in gross oxygen flux between base and tip under light conditions in this species. Hence, we cannot exclude potential differences in light availability created by the protuberances that might affect species photosynthesis.

Also noteworthy were the differences in oxygen fluxes under light conditions, when comparing protuberance tips and bases. *Lithothamnion crispatum* and *M. erubescens* exhibited lower oxygen fluxes at the bases, compared to the respective protuberance tips (Fig. 4a, c), while those in *L. atlanticum* were not different between tips and bases, but significantly higher than in the other two species (Table 1). This observation, together with the higher daily pH fluctuation at the algal thallus surface expressed by *L. atlanticum* (~ 0.4–1.2 pH units), compared to the other two species (~ 0.1–0.6 pH units), suggest potential differences between the species regarding inorganic carbon use strategies and/or carbon-concentrating mechanisms.

Many studies have focused on the acclimation/adaptation potential of coralline algae to environmental changes, such as OA, in order to predict future trajectories of communities and ecosystems^25,43–46^. Yet, only a few have considered the specific role of morphology in species’ OA responses^29,43^. In this regard, light-dependent changes in pH_DBL_ at the algal thallus surface, reported previously in other coralline algae^28–31,47^, have led to the ongoing discussion about whether these changes may ameliorate OA impacts during the day and/or increase them during nighttime^22,29,30,40^. Thus, it could be hypothesized that the strong effect of the rhodolith morphological features on algal surface pH dynamics found in the present study might result in species-specific susceptibility to OA. However, the species’ response to the short-term OA exposure did not show a direct relationship with their morphology and associated DBL dynamics. Yet, the clear differences in the magnitude of daily pH fluctuations at the algal surface the rhodolith species are regularly exposed to indicate that their responses under longer-term exposure to lower seawater pH will most likely differ. This is based on previous suggestions and evidence that species which encounter habitually strong daily pH fluctuations may either be better adapted to cope with decreasing mean seawater pH due to higher phenotypic plasticity^29,48,49^ or be at a disadvantage^47,50^. Furthermore, the lower pH condition did not have any effects on rhodolith light calcification, but induced a strong decline in dark calcification in all species (Fig. 6d–f). The former is not surprising as the short-term experiment did not allow accounting for potential species-specific acclimation capacities that might be expressed under longer term exposure^12,21,23,46^. Dark calcification, on the other hand, is considered as a metabolically independent process, resulting from a combination of purely physical CaCO_3_ precipitation and belated biological activity after a passage from light to dark^51–53^. Thus, it can be assumed that it is independent from acclimation processes and hence, the here found increase in night-time CaCO_3_ dissolution in all three species will most likely be sustained also under longer-term OA conditions. This agrees with previous findings, demonstrating that the main OA effect on calcareous macroalgae is the impact on nighttime rather than on light calcification, often resulting in dissolution and decreased daily net calcification^32,43,45,54–58^. In this context, other studies have also shown that in some species the increased dissolution under long-term OA exposure is off-set by enhanced light calcification^55,56^. Longer-term OA experiments are required to determine if this will be the case in the studied rhodolith species and to further understand the potentially beneficial or detrimental effects of the morphologically related daily pH fluctuations, in order to accurately predict and quantify OA impacts on these organisms and the ecosystems they build.

In summary, understanding the importance of physical and biological factors for coralline algal physiology is key for comprehending the large variability of reported species’ metabolic and calcification rates and to accurately predict the effects of environmental changes, i.e. those related to the ongoing climate change, on different species and the habitats they build or are part of. With the present study, we contribute to a greater understanding of the factors that regulate rhodolith calcification, showing that in addition to species’ physiological traits and environmental drivers, rhodolith morphology represents a critical factor determining daily pH dynamics at the algal surface and consequently, species’ light, dark and net calcification rates (Fig. 7) and potentially their responses to OA. In fact, morphology might be one of the key factors for the commonly found species-specific differences among free-living coralline algae, not only concerning light calcification rates, but especially CaCO_3_ precipitation in the dark (e.g., lower to negative calcification rates in darkness)^8,12,43,53,55,59^.

## Material and methods

### Species description and sample collection

*Lithothamnion crispatum* (Hauck, 1878) and *Melyvonnea erubescens* ((Foslie) Athanasiadis & D.L. Ballantine 2014) are two rhodolith species that can be found throughout the Brazilian coast in the Southwest Atlantic^6^, while *Lithophyllum atlanticum* (T. Vieira-Pinto, M.C. Oliveira & P.A. Horta 2014) is a recently described new species, so far reported only for the Southern Brazilian coast (Rio Grande do Sul, Santa Catarina and Paraná)^37^. These species, which express morphological differences related to the length and diameter of the protuberances (Fig. 1), occur in the majority as monospecific rhodoliths that dominate the rhodolith bed community in the southernmost Brazilian rhodolith bed within the Arvoredo Marine Biological Reserve (− 27°16′25.8", -48°22′0.99")^6,7^.

Monospecific specimens of each of the species were collected in February (Austral summer) from 8 m depth by SCUBA diving. Immediately after collection, samples were placed in coolers with seawater and transported to the laboratory, where samples were dried for computer tomography analyses (n = 5 per species). The remaining rhodoliths were kept in tanks (V = 100 L) with circulating seawater, at 24 °C (temperature observed during sample collection) and under natural light conditions, adjusted to the mean summer light level at collection depth (~ 26% of incident light; K_d_ = 0.19 m^-1^)^60^ for laboratory experiments.

### Characterization of rhodolith morphology

For a quantitative morphological comparison, 3-D images of the rhodoliths were obtained, using the X-Ray Microtomography (Micro-CT) scanning technique^61^. So far, CT-scans have been used in living and fossil rhodoliths to study the associated fauna (internally and externally)^62–64^, rather than to calculate rhodolith surface area and volume. On the other hand, this technique is widely used in other organisms, such as corals, to virtually reconstruct morphological structures and provide high accuracy measurements of the actual surface area^65^.

Micro-CT acquisition of three-dimensional rhodolith images (n = 5 per species) was performed with a Zeiss/Xradia CT scanner, model Versa XRM-500 (Carl Zeiss AG, Germany). The 3-D images were rendered for visualization with Drishti software^66^, ParaView^67^ and Fiji^68^, and ImageJ software^69^ was used for image analysis and quantification. Median and Unsharp Mask filters were applied to images before the segmentation process. After segmentation, surface area and volume of the samples were determined.

### Microelectrode measurements

The influence of rhodolith morphology on diffusive boundary layer (DBL) thickness and potential creation of different microenvironments at the thallus surface was determined using microsensors to measure oxygen and pH dynamics at the protuberance tips and in the gaps between protuberances (“protuberance base”). For this, O_2_ microsensors were custom-made and used as described by Revsbech^70^ and liquid ion exchange (LIX) pH microelectrodes were constructed according to de Beer et al.^71^. Before measurements, the O_2_ microoptode was calibrated with 0 and 100% air saturated seawater obtained by bubbling with gaseous nitrogen and compressed air, respectively. The pH microelectrode was calibrated using pH_NST_ 7.0 and 9.0 buffers (Fluka Analytical, Buchs, Switzerland). The oxygen and pH electrodes had average tip diameters between 10 and 20 µm, and therefore minimal influence on the DBL of the sampled coralline algae.

The experimental set-up used for microprofiling was as described in Hofmann et al.^28,31^, using a flow chamber in which the rhodoliths were exposed to a water flow of approximately 2 cm s^-1^ (judged by particle movement), corresponding to the flow velocity recorded at the sampling site and depth^60^. pH and O_2_ microelectrodes were used to measure depth microprofiles from the surface of the algae into the overlying seawater under light and dark conditions. Microprofiles of the three species were measured under steady state conditions on three individuals from each species at multiple protuberance tips and bases (2–3 per individual).

Microprofiles of O_2_ and pH were obtained by first positioning the sensors at the surface of the algal specimen, using a micromanipulator (Pyroscience, Aachen, Germany), and the aid of a stereomicroscope. Using the software Profix (Pyroscience, Aachen, Germany), the microelectrodes were then programed to run two consecutive profiles from the thallus surface (position 0) into the overlying water column (position 600 µm) at 25 µm intervals within the DBL, and 100 µm intervals outside the DBL. Microprofiles were firstly conducted under saturating light and steady state conditions, which were achieved within one minute. Microprofiles in the dark were then obtained following a dark acclimation period for several minutes, after which there was no noticeable change in the sensor signal. DBL thickness was calculated from the oxygen profiles according to Jørgensen and Revsbech^72^, using the extrapolation of the linear gradient to the bulk seawater concentration. Oxygen fluxes were calculated from the concentration profiles according to Fick’s Law^28,31^ using the diffusion coefficient of 2.01 × 10^–9^ m^2^ s^-1^ for seawater. Differences in pH were calculated by subtracting the surface pH (pH_S_) from the overlying bulk seawater pH (ΔpH = pH_S_–pH_B_).

### Species’ metabolic and calcification rates and responses to short-term exposure to low seawater pH

Photosynthesis, respiration and calcification rates of rhodoliths (different specimens as for microelectrode studies) were determined at ambient and low pH conditions within two days after sample collection. For this, rhodoliths of similar size were incubated individually at 24 °C with filtered seawater (0.45 µm) in sealed custom-made plexiglass chambers (V = 150 mL) with internal circulation provided by a magnetic stirrer. During the incubations, the rhodoliths (n = 5 per species) were exposed for 30 min to a saturating light intensity of 400 µmol quanta m^-2^ s^-1^ (~ 3I_k_- saturating light intensity for photosynthesis; Schubert, unpublished data) for photosynthesis and light calcification measurements and subsequently for another 30 min in darkness to determine respiration and dark calcification rates. Previous tests assured that 30 min incubations provide a high enough signal to noise ratio to allow detection of changes in both oxygen concentration and alkalinity, while ensuring that pH changes during incubations were < 0.1 units.

Incubations were performed either at an ambient pH_NBS_ of 8.12 ± 0.003 (Total alkalinity, A_T_ = 2378 ± 1 µmol kg^-1^, Ω_calc_ = 6.0 ± 0.03) or a low pH_NBS_ of 7.698 ± 0.002 (A_T_ = 2377 ± 1 µmol kg^-1^, Ω_calc_ = 2.7 ± 0.01). The low pH was achieved by bubbling seawater with CO_2_ until the pH had stabilized at the target value, monitored with a pH meter (Metrohm, Switzlerland).

At the beginning and end of each incubation, the oxygen concentration was measured with an oxymeter (YSI 5000–115, Yellow Springs, USA) and the changes in oxygen were corrected afterwards by measurements of control incubations (without rhodolith samples) to account for potential oxygen changes in the incubations water, e.g. due to microbial activity. Also, at the beginning and end of each incubation water samples were taken, poisoned with HgCl_2_ and stored in borosilicate tubes (two tubes per incubation chamber, V = 12 mL each) for later determination of calcification rates. Calcification rates were determined from alkalinity measurements of seawater samples taken before and after the incubation period, using the alkalinity anomaly principle based on the ratio of two equivalents of A_T_ per each mole of CaCO_3_ precipitation^73^. For A_T_ measurements, duplicate analyses of each sample were performed, using the Gran titration method^74,75^. The samples were titrated with HCl 0.1 M and the pH and temperature were recorded using a pH meter (Metrohm, Switzlerland). For quality control, a certified reference material of known total alkalinity was used to calibrate the method (batch 160, supplied by the Marine Physical Laboratory, Scripps Institution of Oceanography, USA), yielding A_T_ values within 5 µmol kg^-1^ of the certified value.

After the experiments, rhodoliths were dried for 48 h at 60 °C and their surface area (used to normalize metabolic and calcification rates) was determined by the wax dipping method^76^ (see Supplementary Fig. S3 online for a detailed description). This method was used instead of the more time-expensive X-ray CT scans, as it represents a fast and cost-effective method, providing results that have been shown to be generally in good accordance with those obtained by CT-scans^77,78^. The consistency of the data obtained by the two methods was verified by comparing the SA/DW ratios (considering dry weight as a factor unaffected by the choice of method), which did not yield significant differences between methods (see Supplementary Fig. S3d online).

### Statistical analysis

Statistical analyses were performed using the software STATISTICA 7.0 (StatSoft, Inc., U.S.A.). Two-way ANOVAs were used to evaluate potential differences in measured parameters between species and locations on the algal thallus (protuberance tip vs. base) and between species and different seawater pH-treatments. One-way ANOVAs were performed to test for differences between species regarding morphology-related parameters (SA/V, SA/DW) and metabolic and calcification rates. Newman-Keuls Significant Difference post-hoc tests were used to identify the statistically different groups. Data were tested a priori for normality and heteroscedasticity, using the Shapiro–Wilk and Levene’s tests, respectively.

## Supplementary Information

Supplementary Information.
