# Author Correction: Calcification in free‑living coralline algae is strongly influenced by morphology: Implications for susceptibility to ocean acidification

**DOI:** 10.1038/s41598-021-94089-5

**Published:** 2021-07-13

**Authors:** Nadine Schubert, Laurie C. Hofmann, Antonella C. Almeida Saá, Anderson Camargo Moreira, Rafael Güntzel Arenhart, Celso Peres Fernandes, Dirk de Beer, Paulo A. Horta, João Silva

**Affiliations:** 1 CCMAR ‑ Center of Marine Sciences, University of Algarve, Campus Gambelas, 8005‑139 Faro, Portugal; 2 Phycology Laboratory, Botany Department, Center for Biological Sciences, Federal University of Santa Catarina, Campus Trindade, Florianopolis, 88010‑970 Brazil; 3 Microsensor Group, Max Planck Institute for Marine Microbiology, Celsiusstrasse 1, 28359 Bremen, Germany; 4 Graduate Program in Oceanography (PPGOCEANO), Center for Physical and Mathematical Sciences, Federal University of Santa Catarina, Campus Trindade, Florianopolis, 88010‑970 Brazil; 5 Porous Media and Thermophysical Properties Laboratory (LMPT), Mechanical Engineering Department, Federal University of Santa Catarina, Campus Trindade, Florianopolis, 88010‑970 Brazil; 6 Present Address: Marine Aquaculture Group, Alfred Wegener Institute Helmholtz Center for Polar and Marine Research, Am Handelshafen 12, 27570 Bremerhaven, Germany; 7 Present Address: Institute of Oceanological Research (IIO), Autonomous University of Baja California, Km 106. Carretera Tijuana‑Ensenada, 22860 Ensenada, Baja California Mexico

Correction to: *Scientific Reports* 10.1038/s41598-021-90632-6, published online 27 May 2021

The original version of this Article contained an error in the Funding section.

“NS was funded by the European Union’s Horizon 2020 research and innovation programme under the Marie Sklodowska-Curie grant agreement (No. 844703). NS and JS received Portuguese national funds from FCT—Foundation for Science and Technology through project UID/Multi/04326/2019. PAH was supported by grants from FINEP/Rede CLIMA (01.13.0353–00) and CNPq-Universal (426215/2016–8). LCH was funded by the National Science Foundation Ocean Sciences International Postdoctoral Research Fellow program (Grant No. 1521610). The work was supported by the DFG grant HO 5439/2–1 within the Initiation of International Collaboration Program to LCH.”

now reads:

“NS was funded by the European Union’s Horizon 2020 research and innovation programme under the Marie Sklodowska-Curie grant agreement (No. 844703). NS and JS received Portuguese national funds from FCT—Foundation for Science and Technology through project UIDB/04326/2020. PAH was supported by grants from FINEP/Rede CLIMA (01.13.0353–00) and CNPq-Universal (426215/2016–8). LCH was funded by the National Science Foundation Ocean Sciences International Postdoctoral Research Fellow program (Grant No. 1521610). The work was supported by the DFG grant HO 5439/2–1 within the Initiation of International Collaboration Program to LCH.”

The original Article has been corrected.

