# Supplementary material for: Calcification in free-living coralline algae is strongly influenced by morphology: Implications for susceptibility to ocean acidification
Source: Sci Rep. 2021 May 27;11:11232. doi: 10.1038/s41598-021-90632-6 (PMC8160205; doi:10.1038/s41598-021-90632-6)
Supplement: Supplementary file 1 — Supplementary Information. [file 41598_2021_90632_MOESM1_ESM.pdf]

SUPPLEMENTARY MATERIAL

Calcification in free-living coralline algae is strongly influenced by morphology: Implications for susceptibility to ocean acidification

Nadine Schubert, Laurie C. Hofmann, Antonella C. Almeida Saá, Anderson Camargo Moreira, Rafael Güntzel Arenhart, Celso Peres Fernandes, Dirk de Beer, Paulo A. Horta, João Silva

Figure S1. Microprofiles of oxygen concentration (left row) and pH (right row) measured with microsensors under light (white symbols) and dark conditions (black symbols) at the protuberance tips and bases of three rhodolith species. Each profile represents an average of three replicate measurements.

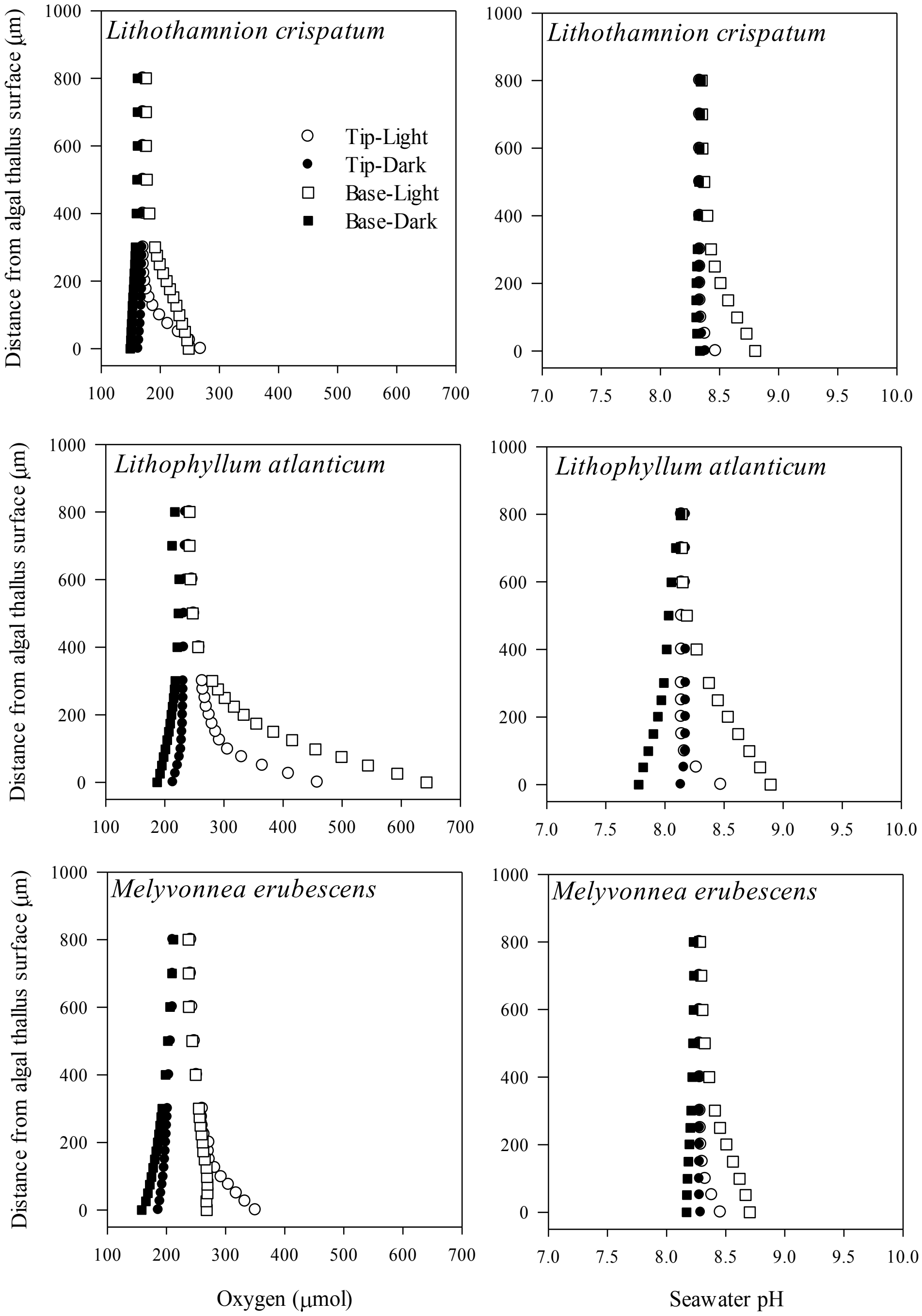

Figure S2. Influence of rhodolith morphology on light microenvironments, measured with (a) a fiber optic light microsensor (Kühl et al. 1994) with a spherical tip (80 µm diameter) connected to a portable spectrometer (USB 4000, Ocean optics, Dunedin, USA) to measure light spectra at the surface of protuberance tip and base (here shown for *Litophyllum atlanticum*). (b) Light reaching the base of the protuberances was calculated by integrating the intensity of the light spectra within the photosynthetically active radiation (PAR) range (400-750 nm) at the rhodolith surface and expressed as percentage of light that reached that area with respect to the tip of the neighboring protuberance (n=3 per species).

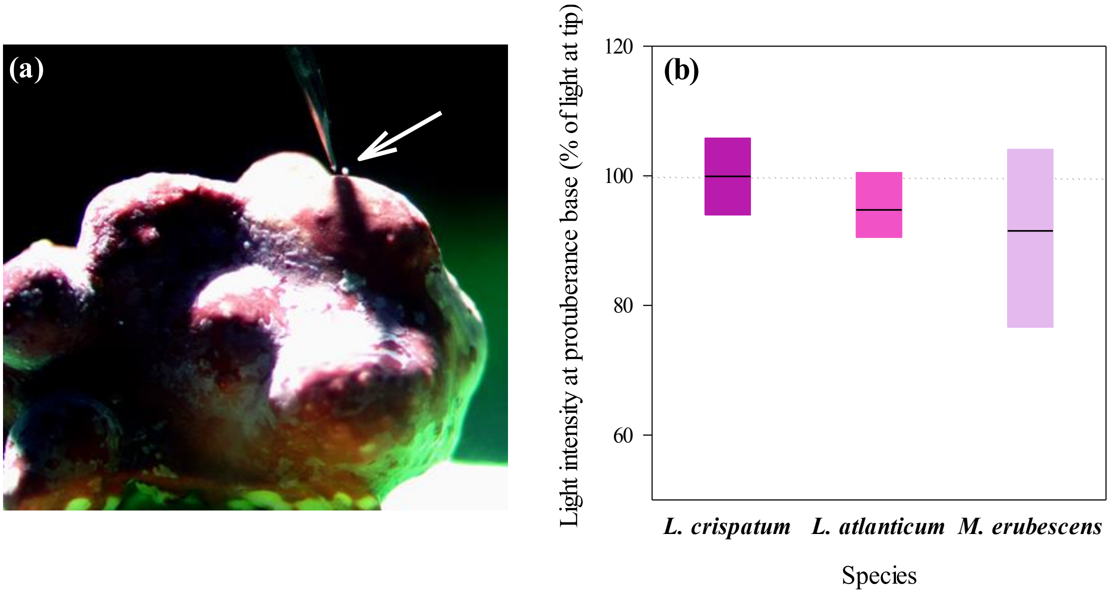

Kühl, M., Lassen, C., & Jørgensen, B. B. (1994). Optical properties of microbial mats: light measurements with fiber-optic microprobes. In Microbial mats (pp. 149-166). Springer, Berlin, Heidelberg.

Figure S3. Surface area determination by the wax dipping method (a-c) and comparison with data obtained by Micro CT-scans (d). Three-dimensional surface area determination by the wax dipping technique, as described for corals by Stimson and Kinzie (1991), was performed on the rhodoliths used in the experiments (n=10 per species). The technique is based on the positive correlation between weight increase of an object when it is dipped in wax and the surface area of the object. Pre-weighted rhodoliths were bound to a nylon string and dipped for two seconds into melted paraffin wax (Merck, paraffin wax pellets) at a temperature of 65°C and afterwards slightly shaken to remove drops (a, b). The increased weight was then converted into surface area, using a calibration factor obtained from a linear regression fit by plotting the increase in mass of objects with known surface area due to the surface coat of wax against their surface area (c). The consistency of the data with those obtained by Micro CT-scans was verified by comparing SA/DW ratios of both methods (t-test, p>0.05), considering dry weight as a factor unaffected by the choice of method (d).

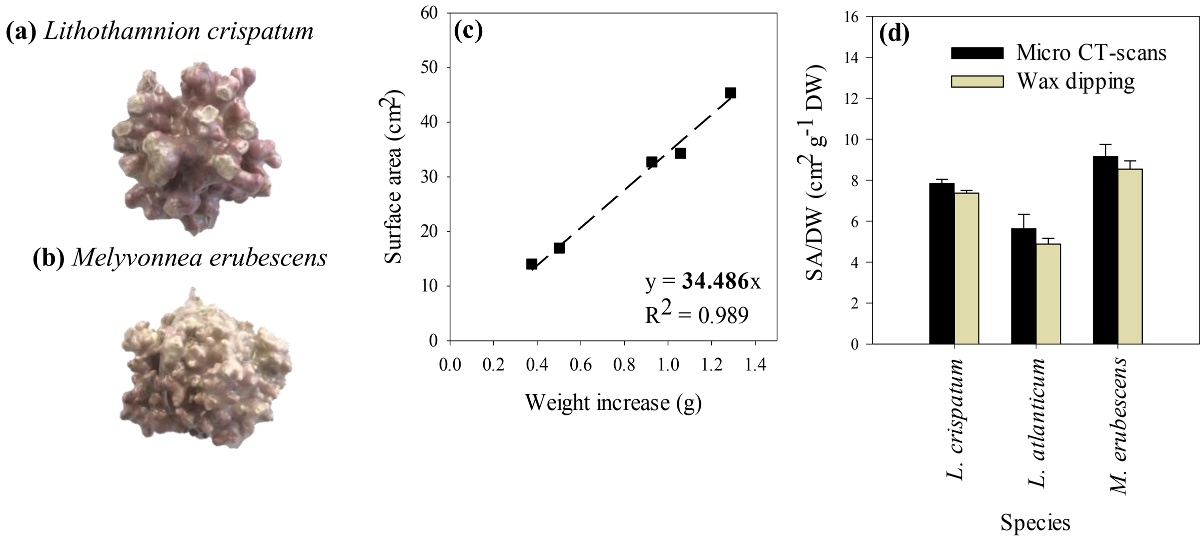

Stimson, J., & Kinzie III, R. A. (1991). The temporal pattern and rate of release of zooxanthellae from the reef coral *Pocillopora damicornis* (Linnaeus) under nitrogen-enrichment and control conditions. *J. Exp. Mar. Biol. Ecol.*, *153*(1), 63-74.
